# The Local Use of Chloral Hydrate

**Published:** 1875-08

**Authors:** 


### The Local Use of Chloral Hydrate.

Dr. Charles A. Peabody, House Surgeon to the City Hospital, at Worcester, Mass., makes the following report of his experience with the local use of chloral. (*Canada Med. Record*, May, 1875.)

I began to use chloral externally about ten months ago in Dispensary practice, experimentally. In this I was associated with Dr. E. Warner, also of the Dispensary staff.

It was first tried in a 5 grain solution, on a small unhealthy ulcer of the leg, with most gratifying result; the dirty unhealthy surface of the sore became clean, healthy granulations sprang up, and the ulcer was soon healed.

After this many ulcers of this kind were treated in this way, and with uniform success, they beginning at once to assume a healthy aspect and soon healing. It was found advisable, however, usually to reduce the strength of the solution to 3 grs. to the ounce of water, after the first two or three days, as it seemed to be then too stimulating.

Encouraged by this success we began to extend its use to chronic eczema, one very aggrevated case of which I have in mind, which was at once much relieved, and within two weeks almost entirely cured. In this case a three grain solution was used from the first, and no other application whatever was allowed.

I have also found it to be, in varying strength, a most excellent application in cases of offensive perspiration and offensive discharge. It has not the powerful and persistent odor of carbolic acid, and is in many cases to be preferred.

In hospital practice the chloral wash has not disappointed my expectations. I have in mind two cases where its good effects were very marked. The first case was an amputation of the thigh, performed for disease of the limb. The wound was dressed with carbolic acid; the flaps did not unite at all, but the cut surfaces assumed, after a few days, an unhealthy look, and became covered with patches of membranous character. Chloral 4 grs. to the ounce was applied, and the very next day all the membranous patches had disappeared, the wound began to look healthy, and granulations were seen springing up over nearly all its surface.

The other case is in hospital now: the foot was amputated through the metatarsal bones for railroad injury. The healing process progressed slowly for a while, and then seemed to come to a stand-still, and for two week no progress whatever could be detected, but the surface of the wound assumed a dirty, unhealthy appearance. Then a 5 gr. chloral wash was applied with immediate good effect. The next day the wound looked healthy, and the process of repair seems now, after three days' use of the chloral, to be fairly started into activity.

Thus, I have briefly indicated the results upon which I base my very favorable opinion of chloral as an external application. Of course, if used indiscriminately and unskillfully, it may disappoint, but it has its place, and if intelligently and judiciously used will not fail, I think, of giving very general satisfaction.

There are a few points worthy of notice in which chloral in solution compares favorably with carbolic acid; these are as follows:

1. It does not have the unpleasant smell of carbolic acid, while it is yet a very excellent deodorizer and antiseptic; it will even, in great measure, deodorize carbolic acid itself.

2. It is a much neater and cleaner dressing than the carbolized oil which is so frequently used.

3. It does not stain or rather fix staines, as carbolic acid does; an important consideration where sheets, etc., are of any value.

4. It does not "kill granulations" as carbolic acid does, but stimulates them.—*Monthly Abstract Med. Sciences.*

